# The effect of sphingosine-1-phosphate on colonic smooth muscle contractility: Modulation by TNBS-induced colitis

**DOI:** 10.1371/journal.pone.0170792

**Published:** 2017-05-11

**Authors:** Aishah Al-Jarallah, Mabayoje Oriowo

**Affiliations:** 1 Department of Biochemistry, Faculty of Medicine, Health Sciences Center, Kuwait University, Jabreya, Kuwait; 2 Department of Pharmacology and Toxicology, Faculty of Medicine, Health Sciences Center, Kuwait University, Jabreya, Kuwait; University of Texas Medical Branch, UNITED STATES

## Abstract

**Aim:**

Increased levels of circulating sphingosine-1-phosphate (S1P) have been reported in ulcerative colitis. The objective of this study was to examine the effect of S1P on colonic smooth muscle contractility and how is it affected by colitis.

**Methods:**

Colonic inflammation was induced by intrarectal administration of trinitrobenzene sulfonic acid. Five days later colon segments were isolated and used for contractility experiments and immunoblotting.

**Results:**

S1P contracted control and inflamed colon segments and the contraction was significantly greater in inflamed colon segments. S1P-induced contraction was mediated by S1PR1 and S1PR2 in control and S1PR2 in inflamed colon segments. S1PR3 did not play a significant role in S1P-induced contractions in control or inflamed colon. S1PR1, S1PR2 and S1PR3 proteins were expressed in colon segments from both groups. The expression of S1PR1 and S1PR2 was significantly enhanced in control and inflamed colon segments, respectively. S1PR3 levels however were not significantly different between the two groups. Nifedipine significantly reduced S1P-induced contraction in control but not inflamed colon segments. Thapsigargin significantly reduced S1P-induced contraction of the inflamed colon. GF 109203X and Y-27632, alone abolished S1P-induced contraction of the control but not inflamed colon segments. Combination of GF 109203X, Y-27632 and thapsigargin abolished S1P-induced contraction of inflamed colon segments.

**Conclusion:**

S1P contracted control colon via S1PR1 and S1PR2 and inflamed colon exclusively via S1PR2. Calcium influx (control) or release (inflamed) and calcium sensitization are involved in S1P-induced contraction. Exacerbated response to S1P in colitic colon segments may explain altered colonic motility reported in patients and experimental models of inflammatory bowel disease.

## Introduction

Inflammatory bowel diseases (IBD) such as ulcerative colitis and Crohn’s disease are characterized by chronic inflammation of unknown etiology. IBD involves a complex interaction between genetic and microbial factors and immune responses (recently reviewed in [1]). Abnormal colonic motility characterized by attenuated rhythmic phasic and tonic contractions and enhanced frequency of giant migrating contractions is an important feature of this condition [2–7]. Dysfunctional gastrointestinal motility has been demonstrated in vitro using patients’ samples. For example, Snape and colleagues (1991) reported a decrease in contraction of colon circular muscle strips from patients with ulcerative colitis to electrical stimulation and bethanechol. Similarly, Al-Saffar and Hellstrom have also reported decreased reactivity of inflamed colon segments isolated from patients with IBD to tachykinins [8]. This has been confirmed by various studies in experimental models of colitis [9–17]. Several mechanisms have been proposed to explain the dysfunctional colonic contractility. These include impaired handling of intracellular calcium concentrations resulting from reduced calcium influx either through reduced expression [14, 16, 17] or enhanced nitrosylation [18] of L-type calcium channels, reduced sarco/endoplasmic reticulum ATPase-2 (SERCA2) [10], inhibition of myosin light chain (MLC) phosphorylation and reduced expression of CPI-17, an endogenous serine/threonine phosphatase in smooth muscle cells [16, 19] and impaired protein kinase C-dependent calcium sensitization [9]. Finally inflammation induced alterations in the enteric nervous system, specifically reduced nitric oxide synthase (NOS) immunoreactive neurons, were also implicated [11].The molecular mechanisms behind enhanced frequency of giant migrating contractions resulting in uncontrolled defecation, hemorrhage and thick mucus secretions associated with IBD [3, 5–7], are however less well defined.

Sphingosine-1-phosphate (S1P) is a bioactive sphingolipid that has been implicated in a variety of biological processes including cell proliferation, cell survival, cell migration and adaptive and immune responses [20]. Sphingosine is synthesized from ceramide, a metabolic product of sphingomyeline and is phosphorylated in vivo by sphingosine kinase (SK) 1 or 2 into the S1P [20]. S1P stimulated contractions in vascular smooth muscle [21–24] and non-vascular smooth muscle preparations [25–31]. S1P also induced relaxation of vascular smooth muscles [32, 33]. S1P-induced modulation of smooth muscle contractility has been implicated in some pathological conditions including hypertension [34–36], bladder overreactivity [28, 37] and asthma [26, 29, 38–42]. Even though elevated levels of S1P were reported in patients with IBD and confirmed in experimental models of ulcerative colitis [43], the effect of S1P on colonic smooth muscle in control and colitic rats has not been investigated.

The main objective of this project was to examine the effect of S1P on contraction of rat colon and how this is affected by experimentally induced colitis. The role of sphingosine-1-phosphate receptors (S1PRs) and potential downstream signal transduction pathways were also investigated.

## Materials and methods

### Animals

Rats were obtained from Charles River Research Models and Services. Seventy two male *Sprague-Dawley* rats weighing between 200–250 g were used in this study. The study was approved by Health Science Center, Kuwait University Ethics Committee for animal use. The study was conducted according to the laboratory’s animal care guidelines at Kuwait University, Kuwait in accordance with the international standards of animal care. Rats were maintained at 22°C on 12-hr light/dark cycle (7 am–7 pm) and water and food were available ad libitum. Rats were anesthetized with ketamine (10 mg/Kg) and xylazine (20 mg/Kg). Experimental colitis was induced by intrarectal administration of 2,4,6-trinitrobenzenesulfonic acid (TNBS, 30 mg, Flucka, Gillingham, UK) dissolved in 0.5 ml of 50% ethanol via a catheter inserted approximately 8 cm from the anal margin and administered at a dose of 73.3 mg/Kg. Rats activity, type of stool and body weights were monitored daily post injection. Some animals died the day after TNBS injection. Dissecting the rats suggested perforation as a potential cause of death. About 5% of the animals died prior to the experimental endpoint. The animals were used five days after TNBS administration. In our experience maximal inflammatory response was obtained five days post TNBS treatment. Rats were sacrificed by concussion followed by exsanguination. Thereafter, the abdominal cavity was opened up and approximately 2 cm segments of the terminal colon was dissected and transferred into a petri-dish containing Krebs’ solution at room temperature for physiological experiments or at 4°C for immunoblotting experiments. The tissues were then cleaned and adherent tissues were removed. Colon segments were used immediately for physiological experiments or stored at -80°C for immunoblotting studies. Smaller colon segments were preserved in phosphate buffered saline containing 10% formalin and processed for histological evaluation of colonic inflammation as previously described [9].

### Physiological experiments

#### Preparation of colon segments

After removing all adhering tissues, colon segments were set up longitudinally in Krebs’ solution at 37°C contained in a 25ml organ bath for isometric tension recording. The composition of the Krebs’ solution was as follows: NaCl, 119; KCl, 4.7; NaHCO_3_, 25; KH_2_PO_4_, 1.2; MgSO_4_, 1.2; CaCl_2_, 2.5 and glucose, 11 mM. The solution was continuously gassed with a 5% CO_2_/95% O_2_ mixture. The preparations were allowed to equilibrate under a resting tension of 1.0 g for up to 60 min during which the bath fluid was changed at least once. Isometric contractions were recorded, through dynamometer UF1 transducers, on a Lectromed 4-channel polygraph (MultiTrace 4P). After the period of equilibration, KCl (80 mM) was added to the bath to test for tissue viability. The tissues were washed immediately after the peak contraction has been obtained. The tissues were washed repeatedly over the next 30 min period. The contraction to KCl was repeated every 30 min until two consecutive contractions did not differ by more than 5%. The last contraction to KCl was used as reference against which contractions to S1P were expressed. Thereafter, S1P (1, 10 or 40 μM) was added non-cumulatively to the bath and the contractions were recorded. Once the peak response to any concentration of S1P has been attained, the bath fluid was drained and replaced with fresh Krebs’ solution. At the end of the experiments, the tissues were washed, weighed, frozen and stored at -80°C until analyzed. S1P induced contraction was expressed as a percentage of KCl-induced contraction as mean KCl induced contraction was significantly not different between control and colitic colon segments (Control: 3.87 ± 0.51, Colitic: 3.93 ± 0.59). Similar experiments were performed using the selective S1PR1 receptor agonist, SEW2871. In experiments conducted with kinase inhibitors or receptor antagonists, the appropriate concentration of each antagonist was added to the organ baths and allowed to equilibrate with the tissues for 30 min before adding S1P. Only one antagonist was tested on any given preparation except when the antagonists were used in combination.

### Determination of S1PR expression Levels by SDS-PAGE and immunoblotting

Total membranes were prepared to determine the expression levels of S1PRs. Colon samples were homogenized on ice for 3 minutes in 20 mM Tris-HCl, pH 7.5 containing 2 mM MgCl_2_, 0.25 M sucrose, and 1x protease inhibitors (Sigma Aldrich, Munich, Germany). Homogenates were centrifuged at 3000 x g for 10 minutes at 4°C and supernatants were subjected to another centrifugation step at 100,000 x g for 1 hour at 4°C. The pellet was suspended in 50 mM Tris-HCl, pH 7.5 containing 1x protease inhibitors cocktail (Sigma Aldrich, Munich, Germany) and 0.1% sodium dodecyle sulfate. Protein concentration was measured using the BCA-protein determination kit (Themoscientific, Ottawa, Ontario, CA) and samples were stored at -80°C for further analysis.

After boiling, samples from control and colitic colon segments (50 μg protein) were subjected to SDS-PAGE as previously described [44]. The membranes were immunoblotted with rabbit anti-S1PR1 (Cayman Chemical, MI, USA), rabbit anti-S1PR2 (Sigma Aldrich, Munich, Germany), rabbit anti-S1PR3 (Cayman Chemicals, MI, USA) or rabbit anti-actin (Abcam, MA, USA). HRP-conjugated donkey anti-rabbit antibody (Jackson ImmunoResearch, PA, USA) was used as secondary antibody. Bands were detected using Super Signal Western Pico chemiluminescence Substrate (Thermoscientific, Ottawa, Ontario, CA) and quantified using Image J software.

### Drugs solutions

The following drugs were used in this study: S1P, SEW2871, W146, JTE-013 and CAY-10444, purchased from Cayman Chemical (MI, USA). Other drugs including nifedipine, thapsigargin, GF 109203X and Y-27632 were purchased from Tocris Bioscience (MN, USA). SEW2871, W146, JTE-013, nifedipine, CAY-10444 and GF 109203X were dissolved in dimethyl sulfoxide. Y-27632 was dissolved in water. S1P was dissolved in pre-warmed solution of bovine serum albumin (4 mg/ml) and incubated at 37°C for 30 min with vortexing in between. This was followed by sonication for 10 sec in a water bath sonicator.

### Statistical analysis

Data was analyzed using the Student’s t-test (Microsoft Excel), represented as mean ± standard error of the mean (SEM) and was considered statistically significant when P < 0.05.

## Results

### Characterization of experimental colitis

**Fig 1:**
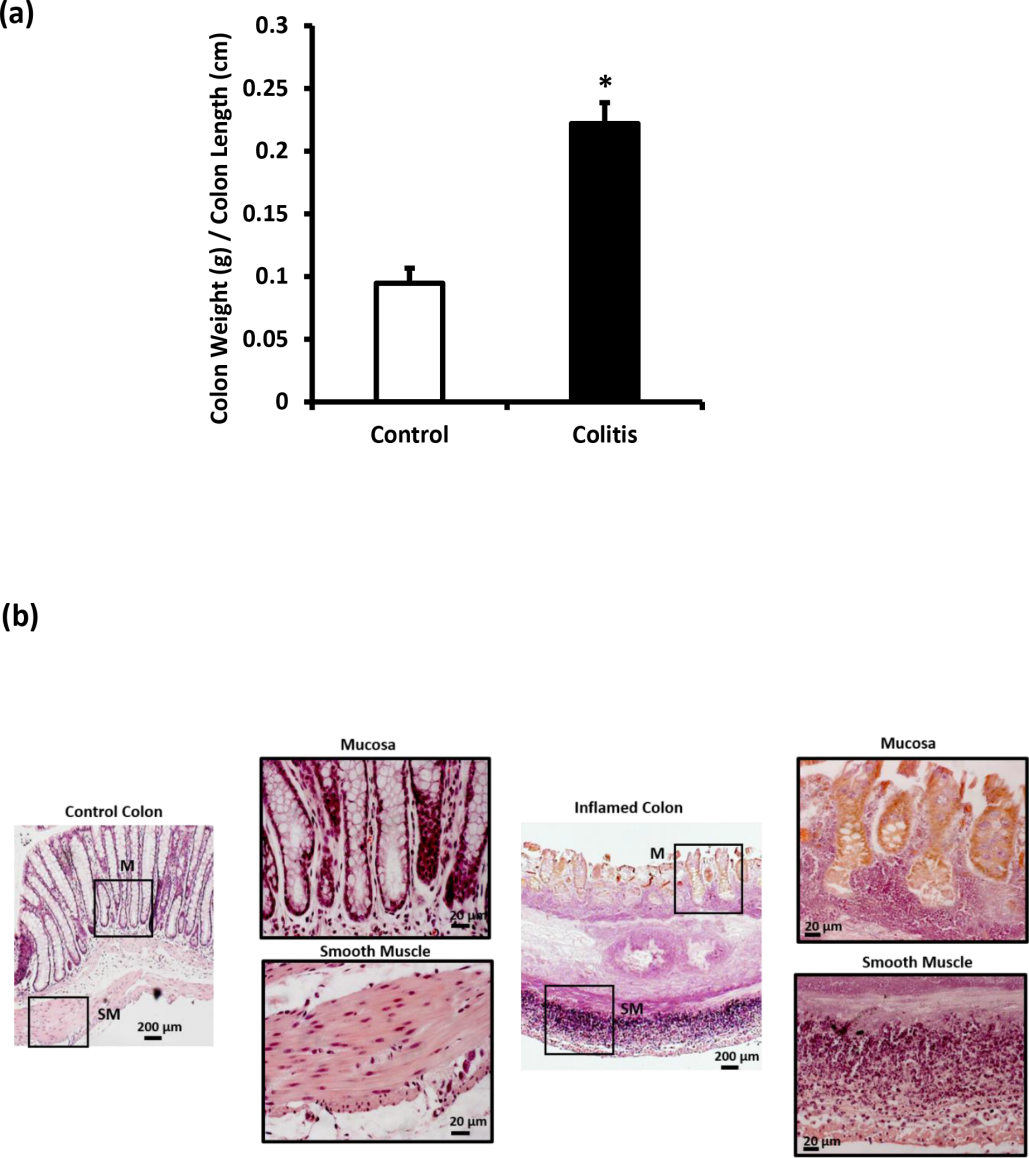
Characterization of ulcerative colitis. (A) Colon weight to length ratio in control and colitic rats. (B) Representative eosin and hematoxylin stained colon sections from control and TNBS-treated rats. Data is mean ± SEM, *P < 0.05.

Rats treated with TNBS weighed significantly less than control rats on day 5 post TNBS injection (223±3.84 g versus 240±2.58 g, P < 0.05). Colon segments from TNBS-treated rats were hypertrophic and weighted significantly (P < 0.05) more compared to colon segments from control rats (Fig 1A).

Macroscopically, colon segments from TNBS-treated rats were grayish thickened, ulcerated and filled with soft feces while colon segments from control rats were, on the other hand, pinkish and engorged with hard feces). Histologically, TNBS treatment resulted in mucosal ulcerations, goblet cells damage and depletion and inflammatory cell infiltration (Fig 1B).

### S1P-induced contractions in control and inflamed colon segments

**Fig 2:**
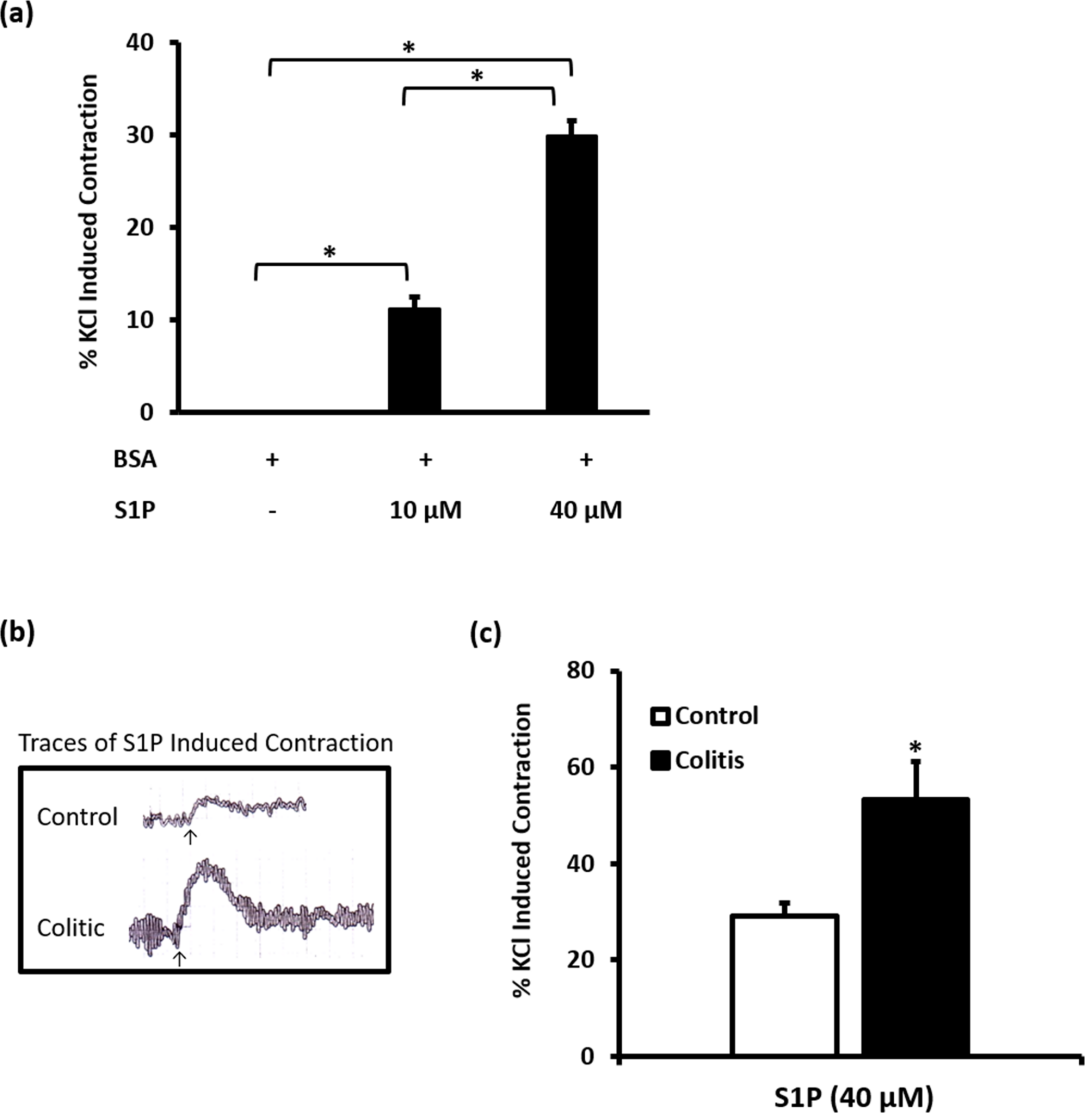
Effect of S1P on KCl-induced contractions in control and colitic colon segments. After equilibration colonic segments from control rats were contracted twice with KCl (80m M) and washed immediately after reaching a peak contraction. Then colonic segments were incubated with BSA (40 μM) or S1P (10 or 40 μM) and allowed to reach a maximal response (A). Traces of S1P (40μM) induced contraction in control and colitic colon segments are shown in (B), arrows indicate S1P addition. Mean S1P (40μM) induced contraction in control and colitic colon segments is shown in (C). Data is mean ± SEM of S1P induced contraction expressed relative to the second KCl-induced contraction, * P < 0.05.

S1P-induced concentration-dependent contractions of colon segments isolated from control rats (Fig 2A).

**Fig 3:**
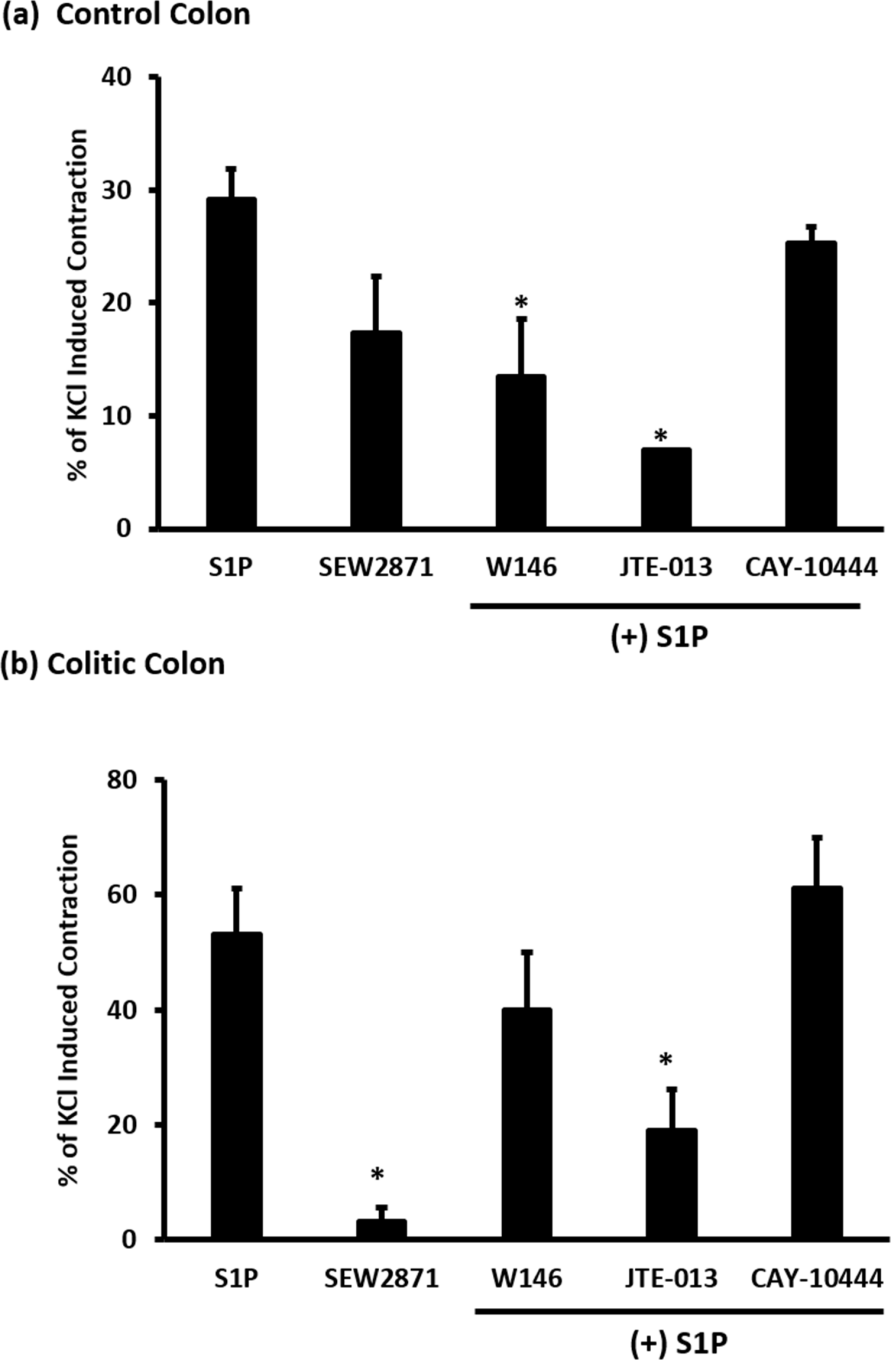
The Role of S1PR in S1P induced contraction in control and colitic colon. Colonic segments from control (A) and colitic (B) rats were equilibrated in Krebs solution for 30 min and contracted twice with KCL (80mM) with consecutive washes after the maximal contraction was reached. Tissues were then preincubated with S1PR1 agonist (SEW2871, 1μM) S1PR1 antagonist (W146, 10μM), S1PR2 antagonist (JTE-013, 1μM) or with S1PR3 antagonist (CAY-10444, 10μM) for 30 min before the addition of S1P (40μM). Data is mean ± SEM of S1P induced contraction is expressed relative to the second KCl-induced contraction, * P < 0.05 relative to S1P induced contraction.

However, we were not able to attain the maximum response as this would involve a lot of S1P. We therefore chose to use 40 μM, a concentration that gave 30% contraction relative to KCl, for subsequent experiments. As shown in (Fig 2B and 2C), S1P (40 μM) induced contractions that were significantly (P < 0.05) greater in colon segments from TNBS-treated rats compared to similar preparations from control rats (Fig 2B). S1PR1 selective agonist, SEW2871, induced contractions in colon segments from control rats (Fig 3A).

SEW2871 however failed to induce a contraction in colon segments from TNBS-treated rats (Fig 3B). Consistent with this finding, the selective S1PR1 antagonist, W146, significantly (P < 0.05) reduced S1P induced contractions in colon segments from control but not TNBS-treated rats (Fig 3A and 3B). In contrast the selective S1PR2 receptor antagonist, JTE-013, significantly (P < 0.05) reduced S1P induced contraction in colon segments from control and TNBS-treated rats by 75% and 60% respectively (Fig 3B). CAY-10444, a selective S1PR3 receptor antagonist had no significant effect on S1P-induced contractions in colon segments from control or TNBS-treated rats (Fig 3A and 3B).

### Immunoblotting experiments

**Fig 4:**
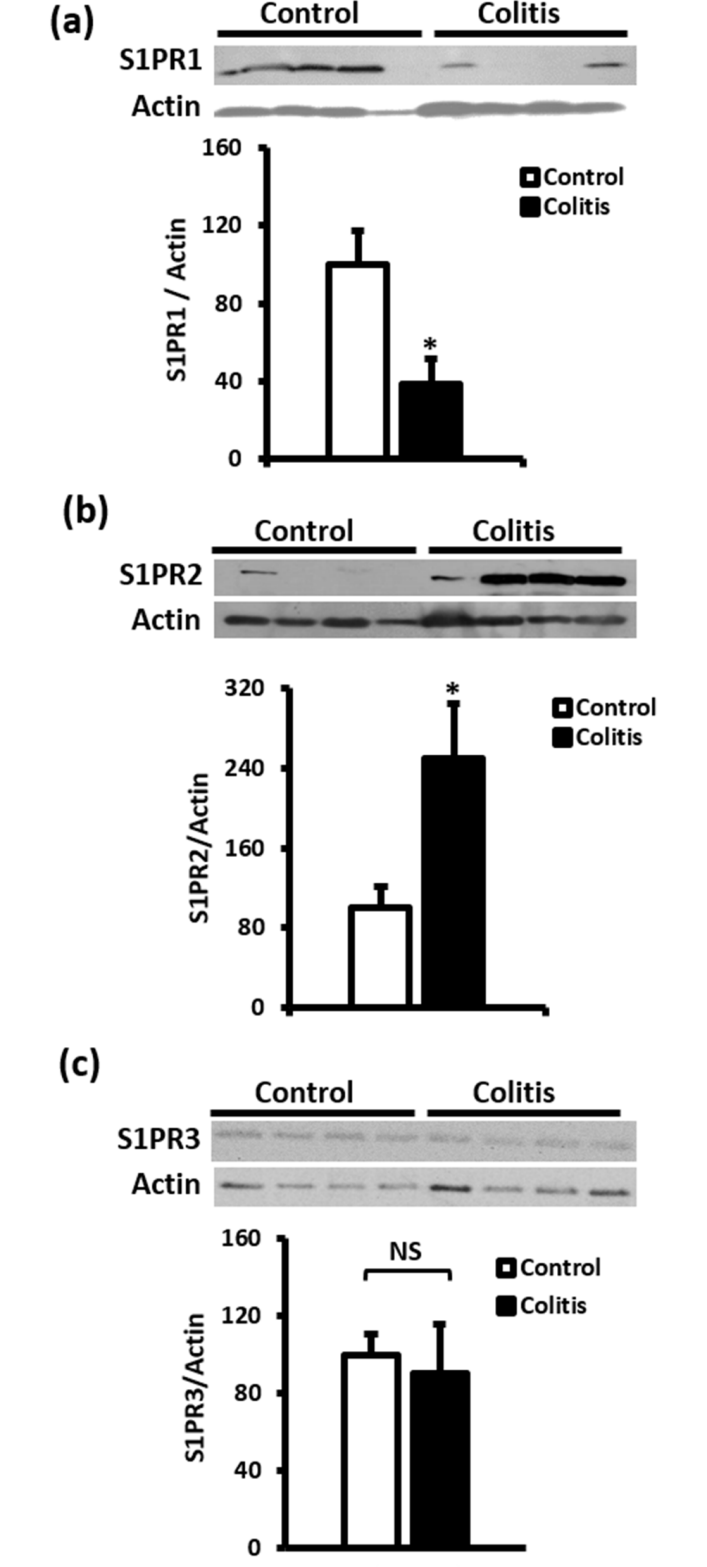
Immunoblotting of S1PR expression in control and colitic colon segments. Representative images of total membrane preps (50 μg protein) from control and colitic colon tissues, from 8–12 independent rats, that were probed with S1PR1 (a), S1PR2 (b) and S1PR3 (c) antibodies. Band intensities were normalized to actin and expressed relative to the controls. Data is mean ± SEM, * P < 0.05 and NS indicates no significance.

As shown in (Fig 4), S1PR1, S1PR2 and S1PR3 receptor proteins are expressed in colon segments from control and TNBS-treated rats.

S1PR1 was expressed at significantly (P < 0.05) higher levels in colon segments from control rats relative to colon segments from TNBS-treated rats (Fig 4A). S1PR2 protein levels were on the other hand significantly (P < 0.05) higher in colon segments from TNBS-treated rats (Fig 4B) whereas S1PR3 expression levels were similar in colon segments from control and TNBS-treated rats (Fig 4C).

### Role of calcium and calcium-sensitization pathways in S1P-induced contractions

**Fig 5:**
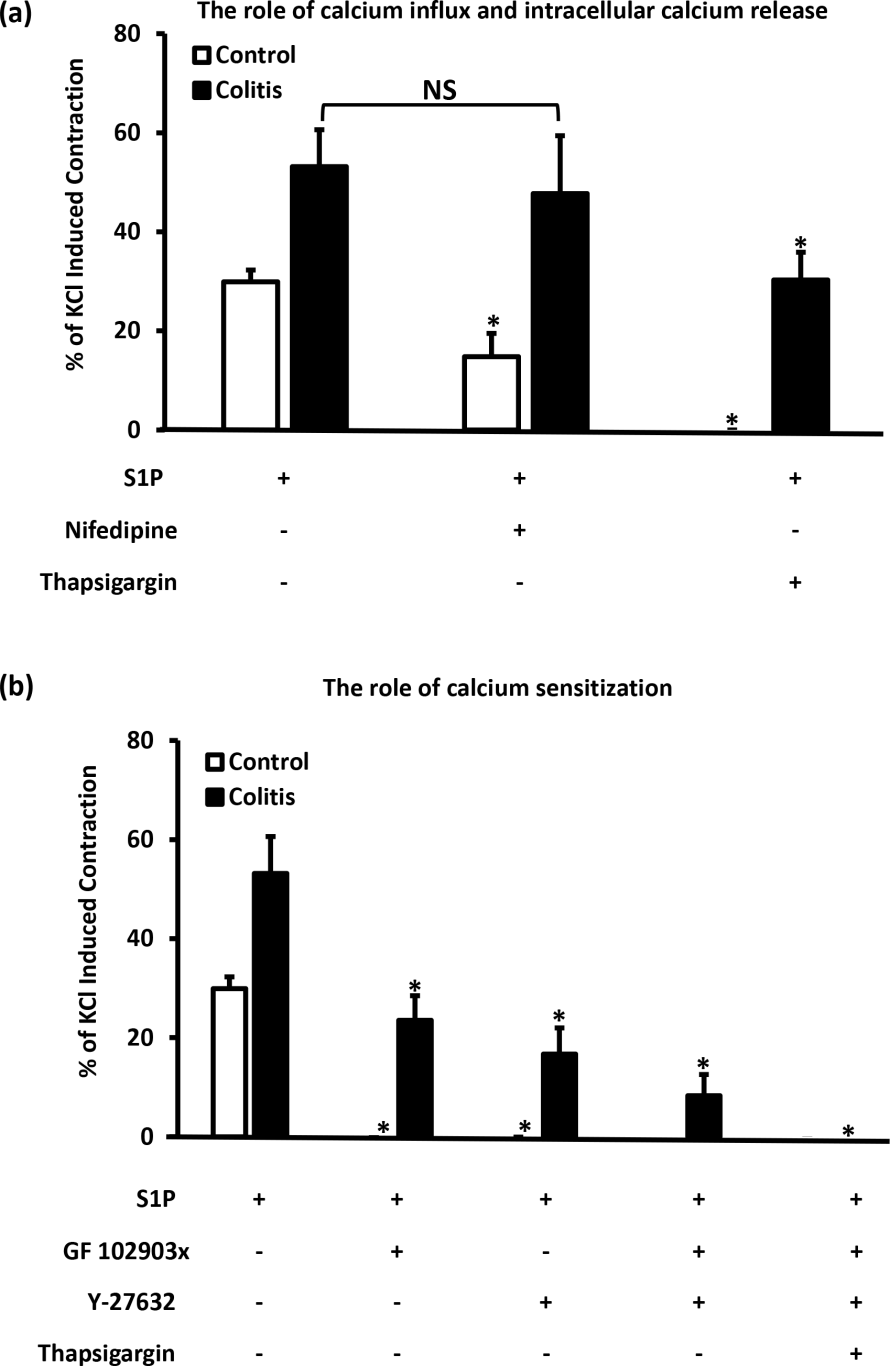
The Role of calcium and calcium sensitization pathways in S1P induced contraction in control and colitic colon. Colonic segments from control (n = 3–6) and colitic (n = 3–7) rats were equilibrated in Krebs solution for 30 min and contracted twice with KCL with consecutive washes after the maximal contraction was reached. Tissues were then preincubated with L-type calcium channel blocker (nifedipine, 1μM) or SERCA antagonist (thapsigargin, 10μM) (A) or with PKC antagonist (GF 102903x, 1μM), ROCK inhibitor (Y-27632, 10μM) or inhibitor cocktails (B) for 30 min before the addition of S1P (40μM). Data is mean ± SEM of S1P induced contraction expressed relative to the second KCl-induced contraction, * P < 0.05 versus S1P induced contraction in corresponding group.

Nifedipine (1 μM), a concentration that abolished KCl-induced contraction [45], significantly (P < 0.05) reduced but did not abolish S1P induced contraction of colon segments from control rats (Fig 5A).

The same concentration of nifedipine did not however affect S1P-induced contractions of colon segments from TNBS-treated rats (Fig 5A). Thapsigargin (10 μM) abolished S1P-induced contraction of colon segments from control rats and significantly (P < 0.05) reduced S1P-induced contraction (approximately 60% reduction) of colon segments from TNBS-treated rats (Fig 5A). GF 102903X and Y-27632 were used to investigate the role of PKC-dependent calcium sensitization pathways in S1P-induced contractions of the colon segments. PKC inhibitor, GF 102903X (1 μM) and Rho-kinase inhibitor (Y-27632, 10 μM) abolished S1P-induced contractions of colon segments from control rats (Fig 5B). However, the same concentrations of these inhibitors reduced S1P-induced contractions of colon segments from TNBS-treated rats by 56% and 68%, respectively when used individually. A combination of GF 109203X (1 μM) and Y-27632 (10 μM) reduced S1P-induced contraction by approximately 85% in colon segments from TNBS-treated rats (Fig 5B). The addition of thapsigargin to GF 102903X and Y-27632 however completely abolished S1P-induced contraction of colon segments from TNBS-treated rats.

## Discussion

**Fig 6:**
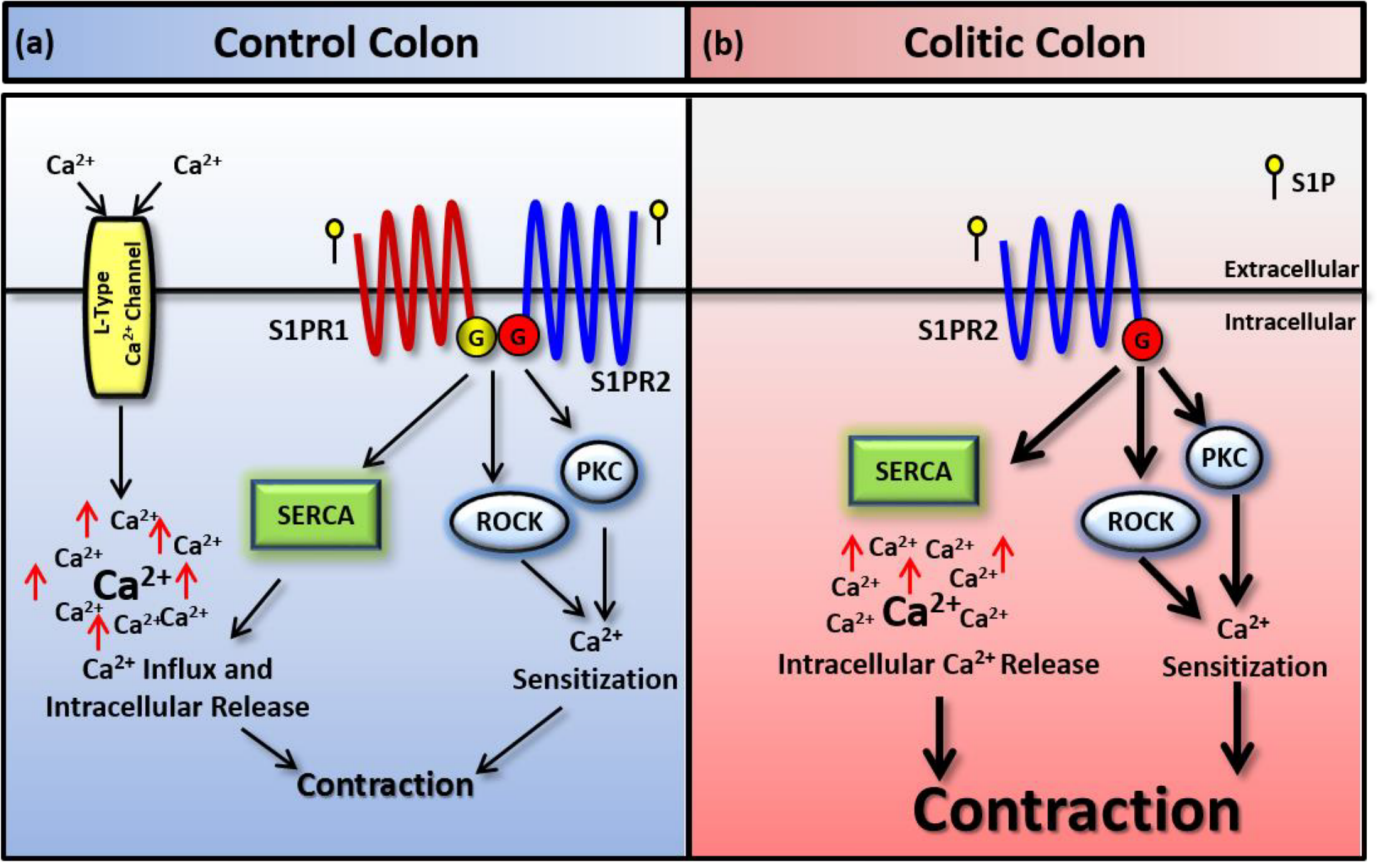
Proposed mechanism of S1P induced contraction in control and colitic colon. In control colon S1P activates S1PR1 and S1PR2 and induce contractions that are dependent on calcium influx via L-type calcium channels, calcium release from the sarcoplasmic reticulum and calcium sensitization pathways including PKC and Rho kinase (A). In colitic colon however S1P induces larger contractions via S1PR2 that are independent of calcium influx through L-type calcium channels yet it involves intracellular calcium release and calcium sensitization pathways (B). The contribution of these pathways however appears to be different in control and inflamed colon segments. Whereas the blockade of a single pathway completely abolishes the response to S1P in control segments, S1P induced contractions appear to be salvaged by other pathways in inflamed colon segments. Eradication of S1P induced contraction in inflamed colon require simultaneous blockade of intracellular calcium release (SERCA) and calcium sensitization pathways (PKC and Rho kinase).

The objective of this study was to test the effects of S1P on colon contractility and test if these effects are modulated by colonic inflammation. The major findings of the study are: 1) S1P induced contractions in control and inflamed colon segments and these contractions were greater in inflamed colon segments. 2) S1P stimulated contractions are mediated by distinct S1PR isoforms in control and inflamed colon segments. 3) Calcium influx through L-type Calcium channels is required for S1P induced contraction in control but not inflamed colon segments. 4) Calcium release from the sarcoplasmic reticulum and calcium sensitization pathways all contributed to S1P induced contractions in control and inflamed colon segments however their contribution differed (Fig 6).

S1P produces its effects by activating G-protein coupled S1P receptors (S1PRs). Of the five S1PRs that have been identified, S1PR1, S1PR2 and S1PR3 receptors are widely expressed in smooth muscles. One or more of these receptors have been reported to mediate the contractile effect of S1P in a variety of smooth muscle preparations. It has been reported that S1P-induced contractions were mediated via S1PR2 receptors in the urinary bladder [28, 31, 37], myometrium [25], airway smooth muscle [26], esophageal [27] and gastric smooth muscle [29, 46]. S1P-induced contraction of gastric smooth muscle is also mediated in part by S1PR1 receptors [29, 46]. There are some reports of S1PR3 receptors mediating S1P-induced contractions in vascular smooth muscle preparations [21, 22, 47]. We therefore sought to determine the receptor(s) mediating the contractile effect of S1P in rat colon. The results showed that in colon segments from control rats, the selective S1PR1 receptor agonist SEW2871 produced a contraction that was not significantly different from S1P suggesting a role for S1PR1 receptor isoform in mediating S1P-induced contraction of the rat colon. This was confirmed by the fact that the contraction was antagonized by W146, a selective S1PR1 receptor antagonist. In addition, S1P-induced contraction of control rat colon was significantly reduced by selective S1PR2 receptor antagonist (JTE-013) but not by the selective S1PR3 receptor antagonist (CAY-10444). Together this suggests that S1P-induced contraction of the control rat colon was mediated via S1PR1 and S1PR2 but not S1PR3 receptor isoforms. This is consistent with the reported involvement of S1PR1 and S1PR2 in mediating S1P-induced contraction of gastric smooth muscle [29, 46]. This result would possibly suggest regional differences in S1PR isoform mediating S1P-induced contraction along the gastrointestinal tract such that S1PR2 receptor mediates contraction in esophageal smooth muscle [27], S1PR1 and S1PR2 receptors in gastric smooth muscle [29, 46] and now in the colon. Interestingly, S1P-induced greater contractions of colon segments from TNBS-treated rats and these contractions were not inhibited by the selective S1PR1 receptor antagonist, W146, at the same concentration that inhibited S1P-induced contraction of colon segments from control rats. This would suggest that S1PR1 receptor did not mediate S1P-induced contraction of inflamed colon segments. In addition, S1P-induced contractions were significantly inhibited by JTE-013 (S1PR2 antagonist) but not CAY-10444 (S1PR3 antagonist). Collectively this data suggests that S1P-induced contraction of inflamed colon segments was mediated by entirely by S1PR2 isoform. These findings were further confirmed by data from immunoblotting experiments. Our results showed that S1PR1, S1PR2 and S1PR3 receptors are expressed in colon segments from control and TNBS-treated rats. S1PR1 protein levels were significantly reduced in colon segments from TNBS-treated rats consistent with lack of involvement of S1PR1 in S1P-induced contraction of inflamed colon segments. On the other hand, S1PR2 protein expression was significantly upregulated in inflamed colon segments which is also consistent with the involvement of S1PR2 in mediating S1P-induced contraction of inflamed colon. Low levels of S1PR3 were detected in control and colitic colon segments however they were not significantly different between the two groups. These finding demonstrate a shift from S1PR1 and S1PR2 in mediating S1P-induced contraction of control colon segments to entirely S1PR2 in mediating S1P-induced contraction of inflamed colon segments. Alterations in the expression levels of S1PR isoforms have been demonstrated in other pathological states for instance upregulation of S1PR1 and S1PR3 and downregulation of S1PR2 were reported in multiple sclerosis lesions [48] and in experimental asthma [49], respectively. The reason behind enhanced response to S1P in inflamed colon segments however remains unclear. S1PR2 was reported to interact with G_i/o_, G_12/13_ and G_q_ [50]. S1PR1 on the other hand is solely coupled to G_i/o_ [50]. S1PR2, via multiple G-proteins, could thus simultaneously activate multiple downstream signaling pathways. Enhanced expression of S1PR2 observed in colitis may therefore result in enhanced activation of downstream signaling pathways and exacerbated response to S1P.

S1P-induced contractions are associated with increased intracellular concentration of calcium resulting from influx of extracellular calcium and/or release from intracellular stores. The contribution of extracellular or intracellular calcium varies between preparations and with the receptor type mediating the S1P response. S1P-induced contraction of porcine retinal arterioles involved influx of extracellular calcium through L-type voltage operated channels [51] with no role for intracellular calcium. On the other hand, S1P-induced contraction of rat cerebellar artery by releasing calcium from intracellular stores [52]. Rosenfeldt and colleagues have shown that S1P-induced contractions of human airway smooth muscle cells is dependent on influx of extracellular calcium [26]. This has been confirmed by [38]. We therefore determined the role of intra- and extracellular calcium in S1P-induced contraction of the rat colon. The results showed that nifedipine, an inhibitor of L-type voltage-gated calcium channels significantly reduced S1P-induced contraction but did not abolish it indicating that S1P-induced contraction of the rat colon was partly mediated via influx of extracellular calcium in agreement with previous reports in the literature [26, 38, 51]. Thapsigargin abolished S1P-induced contraction indicating contribution of intracellular calcium in S1P-induced contractions. Thus both extra- and intracellular calcium are involved in S1P-induced contractions of the rat colon. However, influx of extracellular calcium did not appear to be involved in S1P-induced contraction of inflamed colon segments. This was based on our observation that nifedipine at the same concentration that significantly reduced S1P-induced contraction of colon segments from control rats, had no effect on S1P-induced contraction of the inflamed colon. Previous studies have shown that inflammation impaired influx of extracellular calcium [53–55] possibly due to downregulation of L-type calcium channels in the inflamed tissues [14, 54]. Our results are in agreement with these observations. Thapsigargin significantly reduced S1P-induced contraction of the inflamed colon indicating a role for intracellular calcium in S1P-induced contraction of the inflamed colon.

In addition to increasing intracellular calcium concentration, contractile agonists also sensitize the contractile machinery to calcium. This process of sensitization involves PKC and RhoA/Rho-kinase pathways. According to Chiba et al (2010), S1P-induced contraction of murine bronchial smooth muscle was abolished by Y-27632, an inhibitor of Rho kinase indicating a role for Rho-kinase pathway of calcium sensitization in S1P-induced contraction of the bronchial smooth muscle. The Rho kinase pathway of calcium sensitization has also been implicated in S1P-induced contraction of guinea-pig trachea [38], urinary bladder [31, 37], human airway smooth muscle [26] and porcine retinal arterioles [51]. Kim et al have reported a role for the PKC pathway of calcium sensitization in S1P-induced contraction of feline esophageal smooth muscle cells [56]. Other studies have shown that both PKC and Rho-kinase pathways of calcium-sensitization are involved in S1P-induced contractions of the myometrium [25] and porcine renal arterioles [51]. Our results show that Y-27632 (Rho kinase inhibitor) and GF 109203X (PKC inhibitor) abolished S1P-induced contraction of colon segments from control rats. This would suggest that both PKC and Rho-kinase pathways of calcium-sensitization are involved in S1P-induced contraction of the rat colon. The fact that both inhibitors individually abolished S1P-induced contractions would possibly reflect a cross-talk between the pathways as suggested by Kamiya et al in porcine renal arterioles [51]. S1P could sequentially activate PKC and Rho-kinase pathways of calcium sensitization. Accumulating evidence in the literature suggests the sequential activation of PKC and Rho-kinase pathways of calcium sensitization in smooth muscle cells [57–60]. In inflamed colon however, Y-27632 and GF 109203X significantly reduced S1P-induced contraction, yet did not abolish it. This may suggest reduced contribution of PKC and Rho kinase to S1P induced contraction in inflamed colon. We have previously reported reduced PKC mediated calcium sensitization in response to carbachol in inflamed colon segments [9]. A combination of Y-27632 and GF 109203X did not abolish S1P-induced contraction and left a residual contraction of about 15%. The residual contraction however was completely abolished in the presence of thapsigargin.

It was therefore concluded that S1P-induced contractions of the rat colon via S1PR1 and S1PR2 in segments from control rats but exclusively via S1PR2 in inflamed segments from TNBS-treated rats. The contractions involved multiple signaling pathways including influx of extracellular calcium through nifedipine-sensitive L-type voltage-gated channels, calcium release from intracellular stores and calcium sensitization through PKC and Rho-kinase pathways. Enhanced contractile response to S1P in inflamed colon accompanied with the reported increase in S1P levels may possibly contribute to the reported increase in colonic motility in patients and experimental models of inflammatory bowel disease.

## Supporting information

S1 File Raw data underlying the results. (XLSX)
